# Maternal probiotic milk intake during pregnancy and breastfeeding complications in the Norwegian Mother and Child Cohort Study

**DOI:** 10.1007/s00394-019-02072-8

**Published:** 2019-09-10

**Authors:** Sofiia Karlsson, Anne-Lise Brantsæter, Helle Margrete Meltzer, Bo Jacobsson, Malin Barman, Verena Sengpiel

**Affiliations:** 1 Department of Obstetrics and Gynaecology, Sahlgrenska University Hospital, Gothenburg, Sweden; 2 Division of Infection Control, Environment and Health, Norwegian Institute of Public Health, Oslo, Norway; 3 Department of Obstetrics and Gynaecology, Institute of Clinical Sciences, University of Gothenburg, Gothenburg, Sweden; 4 Department of Genetics and Bioinformatics, Domain of Health Data and Digitalisation, Institute of Public Health, Oslo, Norway; 5 Department of Biology and Biological Engineering, Food and Nutrition Science, Chalmers University of Technology, Gothenburg, Sweden

**Keywords:** Probiotics, Probiotic milk intake, Cessation of breastfeeding, Mastitis, Breastfeeding complications, The Norwegian Mother and Child Cohort Study

## Abstract

**Purpose:**

During the time of breastfeeding, a third of all women contract (or: fall ill in) mastitis—the leading cause of precocious weaning. Recent studies indicate that probiotics intake may prevent mastitis by altering the breast’s bacterial flora. The aim of this study was to examine whether probiotic milk intake during pregnancy is associated with less breastfeeding complications and longer breastfeeding duration.

**Methods:**

This study included 57,134 women, with live singleton term births, participating in the Norwegian Mother and Child Cohort Study. Probiotic milk intake during the first half of pregnancy was self-reported in a validated food frequency questionnaire at gestational week 22. At 6 month postpartum, women reported complications, including mastitis, and duration and exclusivity of breastfeeding. The association between probiotic milk intake and breastfeeding complications and duration was studied by adjusted logistic regression models.

**Results:**

Probiotic milk intake was associated with increased risk for mastitis [adjusted odds ratio (aOR) 1.09, 95% confidence interval (CI) 1.02–1.16] and for any breastfeeding problems during the first month (aOR 1.19, 95% CI 1.10–1.21). However, cessation of predominant (aOR 0.95, 95% CI 0.91–0.96) or any (aOR 0.79, 95% CI 0.75–0.84) breastfeeding earlier than at 4 months was less frequent in probiotic milk consumers than in non-consumers.

**Conclusions:**

Even though probiotic milk intake during the first half of pregnancy was statistically associated with increased risk for breastfeeding complications, including mastitis, the association is probably not causal. Probiotics intake was namely associated with longer breastfeeding duration and there was indication of socioeconomic confounding. Further studies, i.e., large randomized-controlled trials, are needed to understand the association between probiotic intake and breastfeeding complications.

**Electronic supplementary material:**

The online version of this article (10.1007/s00394-019-02072-8) contains supplementary material, which is available to authorized users.

## Background

Lactational mastitis is defined as an inflammatory process of the mammary gland, characterized by pain in the breast in conjunction with flu-like symptoms during breastfeeding. The World Health Organization (WHO) review on lactational mastitis reports an incidence ranging between 2.6% and 33% [1]. While WHO recommends 6 months of exclusive breastfeeding, lactational mastitis is the leading cause of unplanned precocious weaning [1–3]. It causes substantial suffering for the mother and often disturbs the sensitive period of bonding between mother and newborn.

While mastitis previously was considered the consequence of a bacterial infection, new evidence suggests that breast health is instead determined by a balance between different microbiota in the breast tissue, as well as by the state of the host’s immune system [4, 5]. While antibiotics have been the traditional treatment approach, four recent randomized-controlled trials (RCT) from Spain presented promising results with treatment (*n* = 352 [6], *n* = 108 [7]) or prophylactic intake (*n* = 108 [8], *n* = 625 [9]) of certain probiotic strains namely *Lactobacillus (L.) salivarius*, *L. gasseri,* and/or *L. fermentum*.

Several mechanisms for improvement of breast flora by probiotics have been described, e.g., local competitive exclusion [10], production of antimicrobials [11], normalization of breast tissue permeability [12], and increase of immunoglobulin A in breast milk, which may limit the bacteria’s ability to damage mammary epithelium [13].

Today, probiotics are part of many milk products commonly purchased and widely consumed by the general population, including pregnant women. Our group has previously reported that free-market probiotic milk intake during pregnancy containing Lactobacillus acidophilus La-5 (La-5), Bifidobacterium lactis Bb12 (Bb12), and Lactobacillus rhamnosus GG (LGG) is associated with decreased risk for preterm delivery [14] and preeclampsia [15, 16]. To our knowledge, no previous studies have investigated the association between free-market probiotic milk intake and breastfeeding complications and duration. The Norwegian Mother and Child Cohort Study (MoBa) has compiled detailed information on maternal probiotic milk intake during pregnancy, comprehensive information on breastfeeding, as well as general information on health and lifestyle [17]. It is thus a unique source for studying a possible preventive effect of probiotic milk intake during pregnancy on breastfeeding complications and duration in a population-based cohort.

### Objective

We hypothesized that free-market probiotic milk intake during pregnancy prevents breastfeeding complications and thus promotes longer breastfeeding by stabilizing healthy breast flora. The aim of this study was to evaluate whether intake of probiotic milk products during pregnancy is associated with less breastfeeding complications, i.e., mastitis, medication for mastitis, sore nipples or other problems, or associated with longer breastfeeding duration (no cessation of any or predominant breastfeeding before 4 months).

## Materials and methods

### Study population

The MoBa is a prospective, population-based pregnancy cohort study conducted by the Norwegian Institute of Public Health [17]. Participants were recruited from all over Norway from 1999 to 2008, and 41% of invited women consented to participate. Follow-up is conducted by questionnaires at regular intervals and by linkage to pregnancy and birth records in the Norwegian Medical Birth Register (NMBR) [18]. All questionnaires (*Q*) are available on the website of the Norwegian Institute of Public Health [19].

This study is based on version 10 of the quality-assured data files released for research in 2017.

### Inclusion and exclusion criteria

**Fig. 1:**
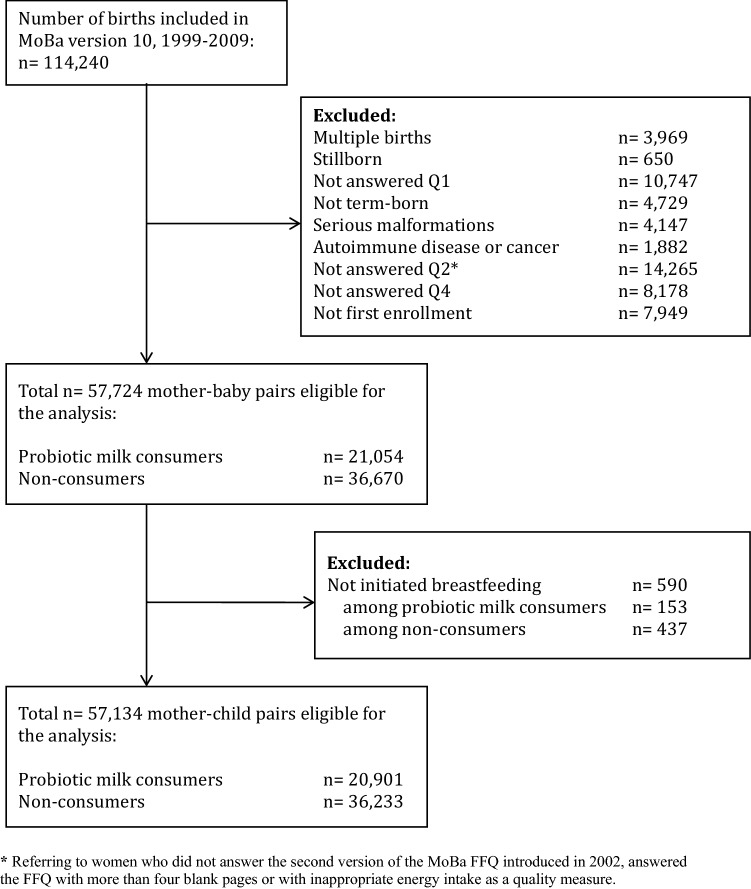
Flowchart showing selection of study participants from the Norwegian Mother and Child Cohort Study (MoBa)

Out of 114,240 births registered in MoBa, all singleton pregnancies with live births after gestational week 37 + 0 were included in the study. Women had to have filled in questionnaires Q1 on general health and lifestyle, Q2 on dietary habits during pregnancy, and Q4 on follow-up 6 month postpartum. As a quality measure of Q2, a food frequency questionnaire (FFQ), only women reporting an energy intake between 4.5 and 20 megajoules (MJ) daily were included. Mothers with reported autoimmune disease or cancer were excluded, as were babies born with serious malformations. Only the first pregnancy enrolled in MoBa was included in the analyses, to avoid repeated assessments of the same mother. After exclusion of women who did not initiate breastfeeding, 57,134 mother–baby pairs remained (see Fig. 1).

### Exposure

Maternal probiotic milk intake during the first half of pregnancy was self-reported in the MoBa FFQ, a semi-quantitative questionnaire designed to record dietary habits. FoodCalc [20] and the Norwegian Food Composition Table [21] were used to calculate food and nutrient intakes. A validation study of the FFQ showed that, relative to a dietary reference method and several biological markers, the MoBa FFQ produces a realistic estimate of habitual intake and is a valid tool for ranking pregnant women according to high and low intakes of energy, nutrients, and food [22].

The FFQ asked specifically about intake frequency of two probiotics-containing dairy products produced by Tine SA, Oslo, Norway (Product A: Biola^®^, containing La-5, Bb12 and LGG*;* and product B: Cultura^®^, containing La-5 and Bb12). Responses ranged from “never” to “8 or more times per day”, with a total of 11 response alternatives. One glass was specified as 250 ml. The bacteria count in these beverages is indicated as a minimum of 2 × 10^8^ CFU of LGG and Bb12 and 2 × 10^7^ CFU of La-5 per 1 mL product A and a minimum of 2 × 10^8^ CFU of Bb12 and 2 × 10^7^ CFU of La-5 per 1 mL product B. These products were the only widely consumed probiotic products available on the Norwegian market at the time of the study. The MoBa FFQ included questions about the use of dietary supplements and an open text field for reporting supplements other than those listed. Very few women (fewer than 0.5%) reported consumption of probiotic supplements, and intake of probiotics from supplements was thus not considered in this study.

Probiotic milk intake from both sources combined was studied as a dichotomous variable (yes/no) as well as in tertiles of probiotics intake (low, medium, and high).

### Outcome

In Q4, administered 6 month postpartum, women were asked whether they had consulted a doctor, midwife, or health visitor during the first month after delivery for breast-related problems, specified as “mastitis”, “sore nipples”, or “breastfeeding problems”, and whether they had received “medication for mastitis”. In addition to these four variables, a combined variable, “any breastfeeding problems”, was created.

Furthermore, mothers reported the specifics of their babies’ nutrition, both during the first week of life, choosing between breast milk, sugar water, water, and different types of formula, and then monthly, choosing between breast milk and different types of formula. Two breastfeeding variables were created based on the WHO definitions [23]: “cessation of predominant breastfeeding before 4 months” and “cessation of any breastfeeding before 4 months”. “Exclusive breastfeeding” could only be reported for the first week of life, as the questionnaires did not specifically ask about ingestion of water, water-based drinks, and fruit juices later on. “Predominant breastfeeding” refers to infants either given only breast milk (exclusive breastfeeding) or breast milk and water-based drinks, but infants should not be fed with solid food, non-human milk or formula. This group thus includes those who were breastfed exclusively during their first week of life. “Any breastfeeding” refers to infants either given only breast milk (exclusive breastfeeding), predominantly breastfed (see above) or partially breastfed, i.e., given solid food, formula, or non-human milk in addition to breast milk [23].

### Confounders

Confounders were selected a priori. Maternal age was registered in the NMBR and used as a continuous variable. The following variables were self-reported in Q1: maternal pre-pregnancy BMI was calculated based on reported height and pre-pregnancy weight and used as a categorical variable (< 18.5, 18.5–24.9, 25–29.9, > 30 kg/m^2^). Maternal education was categorized as < 13, 13–16, ≥ 16 years. Family income was recorded as neither, one or both partners earning > 300,000 Norwegian Crowns (NOK)/year. Maternal smoking categories during pregnancy were never, occasionally, and daily.

Based on the FFQ, daily fibre and energy intake were considered as continuous variables, while non-probiotic yoghurt and milk consumption were calculated as described above for probiotic milk intake.

### Statistics

All analyses were performed using IBM^®^ SPSS^®^ Statistics version 25. Maternal characteristics, related to probiotic milk consumption, were analysed using Pearson’s chi-square test or the two-sided Fisher’s exact test. The Kruskal–Wallis test was used to study whether there was a statistical difference between the amounts of probiotic milk intake in the different maternal characteristic categories.

The associations between being a probiotic milk consumer and breastfeeding complications and duration were studied by logistic regression analysis, unadjusted and adjusted for the confounders described above. Missing data were given a category of their own.

Sensitivity analyses were performed for the association between education and reported breastfeeding complications and duration, as well as between reported breastfeeding complications and breastfeeding duration.

## Results

### Probiotic milk intake in the study population

In our study population, 20,901 (36.6%) women were probiotic milk consumers. The median daily intake (interquartile range) among consumers was 54 ml/day (IQR 22–179).

Probiotic milk consumers had lower parity, although they were older. They had higher educational levels and income as well as more health-conscious behaviour, with higher fibre intake and less smoking than non-consumers (see Table 1).

**Table 1:** Probiotic milk intake according to maternal characteristics, *n* = 57,134 women

	Probiotic milk consumption		*p* value^1^	Mean (SD) daily probiotics intake among probiotics consumers, ml/day	*p* value^1^
	No, number (%)	Yes, number (%)			
All	36,233 (63.4)	20,901 (36.6)		123 (176)	
Maternal age, years					
< 25	4190 (11.6)	1830 (8.8)	< 0.001	111 (168)	< 0.001
25–29	12,278 (33.9)	7193 (34.4)		122 (174)	
30–34	15,478 (42.7)	9217 (44.1)		123 (175)	
≥ 35	4287 (11.8)	2661 (12.7)		135 (185)	
Pre-pregnancy BMI, kg/m^2^					
< 18.5	1025 (2.8)	582 (2.8)	< 0.001	129 (182)	0.01
18.5–24.9	22,526 (62.2)	14,572 (69.7)		124 (174)	
25–29.9	8070 (22.3)	3961 (19.0)		122 (178)	
≥ 30	3686 (10.2)	1339 (6.4)		113 (171)	
Missing	926 (2.6)	447 (2.1)		128 (204)	
Parity					
0	17,061 (47.1)	11,987 (57.4)	< 0.001	126 (178)	< 0.001
1	12,468 (34.4)	5957 (28.5)		117 (171)	
2	5398 (14.9)	2436 (11.7)		119 (173)	
≥ 3	1278 (3.5)	506 (2.4)		135 (196)	
Missing	28 (0.1)	15 (0.1)		71 (125)	
Maternal education, years					
< 13	12,192 (33.6)	4555 (21.8)	< 0.001	128 (186)	0.01
13–16	15,250 (42.1)	9146 (43.8)		119 (174)	
≥ 16	8032 (22.2)	6840 (32.7)		123 (168)	
Missing	759 (2.1)	360 (1.7)		144 (226)	
Family income > 300,000 NOK					
Neither partner	10,678 (29.5)	4756 (22.8)	< 0.001	115 (168)	< 0.001
One partner	15,308 (42.2)	8379 (40.1)		123 (172)	
Both partners	9287 (25.6)	7298 (34.9)		127 (181)	
Missing	960 (2.6)	468 (2.2)		138 (219)	
Smoking in pregnancy					
Never	32,802 (90.5)	19,864 (95.0)	< 0.001	123 (176)	0.002
Occasionally	1025 (2.8)	401 (1.9)		124 (166)	
Daily	2193 (6.1)	540 (2.6)		119 (177)	
Missing	213 (0.6)	96 (0.5)		109 (177)	
Tertiles of daily energy intake					
1st	12,987 (35.8)	6044 (28.9)	< 0.001	85 (107)	< 0.001
2nd	11,948 (33.0)	7111 (34.0)		112 (144)	
3rd	11,298 (31.2)	7746 (37.1)		161 (229)	
Tertiles of daily fibre intake					
1st	13,388 (36.9)	5607 (26.8)	< 0.001	96 (146)	< 0.001
2nd	12,068 (33.3)	7010 (33.5)		117 (168)	
3rd	10,777 (29.7)	8281 (39.6)		146 (196)	
Tertiles of daily non-probiotic milk intake					
1st	11,610 (32.0)	6695 (32.0)	0.29	130 (187)	< 0.001
2nd	13,121 (36.2)	7453 (35.7)		123 (161)	
3rd	11,502 (31.7)	6753 (32.3)		115 (179)	
Tertiles of daily non-probiotic yoghurt intake					
1st	13,958 (38.5)	4967 (23.8)	< 0.001	120 (175)	< 0.001
2nd	11,389 (31.4)	8013 (38.3)		105 (157)	
3rd	10,886 (30.0)	7921 (37.9)		143 (191)	
Caesarean section					
No	31,497 (86.9)	18,255 (87.3)	0.16	122 (175)	0.10
Yes	4736 (13.1)	2646 (12.7)		130 (181)	
NICU admission					
no	31,948 (88.2)	18,134 (86.8)	< 0.001	123 (174)	0.53
yes	4285 (11.8)	2767 (13.2)		122 (185)	
Baby SGA					
No	35,727 (98.6)	20,593 (98.5)	0.46	123 (175)	0.50
Yes	506 (1.4)	308 (1.5)		141 (224)	
Baby LGA					
No	34,772 (96.0)	20,234 (96.8)	< 0.001	123 (176)	0.55
Yes	1461 (4.0)	667 (3.2)		120 (159)	

*IQR* interquartile range, *SGA* small for gestational age, according to Marsál, *LGA* large for gestational age, according to Marsál [24]

^1^*p* value according to Pearson’s chi-square test or two-sided Fisher’s exact test, as appropriate

^2^*p* value according to Kruskal–Wallis test

### Breastfeeding complications and duration

During the first month after delivery, a total of 4675 (8%) women in the study population contacted healthcare services for mastitis and 3127 of these (6% of the total study population) received medication for the mastitis. Sore nipples were reported by 3595 women (6%) and 3665 (6%) reported other breastfeeding problems. In total, 8788 women (15%) contacted healthcare services for any breastfeeding problems during the first month after delivery. Of the study population, 22,235 women (39%) ceased to predominantly breastfeed and 6567 women (11%) stopped any breastfeeding before the baby reached the age of 4 months.

Probiotic milk intake was significantly associated with a higher incidence of breastfeeding complications (except for medication-treated mastitis), as well as a lower frequency of breastfeeding cessation before 4 months. Odds ratios (OR) became less pronounced after adjustment, but results remained significant (except for medication-treated mastitis, see Table 2).

**Table 2:** Associations between probiotic milk intake and breastfeeding complications and breastfeeding duration, *n* = 57,134 women

	Among non-consumers	Among probiotic milk consumers	Unadjusted		Adjusted^a^	
	Number (%)	Number (%)	OR (CI)	*p*	OR (CI)	*p*
Mastitis	2,818 (7.8)	1,857 (8.9)	1.16 (1.09–1.23)	< 0.001	1.09 (1.02–1.16)	0.01
Medication for mastitis	1,892 (5.2)	1,235 (5.9)	1.14 (1.06–1.23)	0.001	1.07 (1.00–1.15)	0.09
Sore nipples	2,029 (5.6)	1,566 (7.5)	1.37 (1.28–1.46)	< 0.001	1.22 (1.14–1.31)	< 0.001
Other breastfeeding problems	2,053 (5.7)	1,612 (7.7)	1.39 (1.30–1.49)	< 0.001	1.22 (1.13–1.30)	< 0.001
Any breastfeeding problems	5,154 (14.2)	3,634 (17.4)	1.27 (1.21–1.33)	< 0.001	1.19 (1.10–1.21)	< 0.001
Cessation of predominant breastfeeding before 4 months	14,466 (39.9)	7,769 (37.2)	0.89 (0.86–0.92)	< 0.001	0.95 (0.91–0.96)	0.006
Cessation of any breastfeeding before 4 months	4,758 (13.1)	1,809 (8.7)	0.63 (0.59–0.66)	< 0.001	0.79 (0.75–0.84)	< 0.001

^a^Logistic regression adjusted for maternal age, maternal pre-pregnancy BMI, maternal education, family income, maternal smoking, fibre intake, energy intake, non-probiotic yoghurt consumption, and non-probiotic milk consumption

However, there was no dose–response association between amount of probiotic milk intake either with breastfeeding complications or duration (see Supplemental Table 1).

Sensitivity analyses were performed to better understand the contradicting results of probiotic milk consumers having a higher incidence of breastfeeding complications, while also breastfeeding longer. First, the known association between breastfeeding complications and earlier cessation of breastfeeding was confirmed in this study population (see Table 3). Associations remained the same when studying the subgroups of probiotic milk consumers and non-consumers separately (data not shown).

**Table 3:** Association between breastfeeding problems and cessation of predominant breastfeeding before 4 months, *n* = 57,134 women

	Predominant breastfeeding before age 4 months (in %) in case of breastfeeding complication		Unadjusted		Adjusted^a^	
	Not present	Present	OR (CI) for cessation of breastfeeding in case of breastfeeding complication	*p*	OR (CI) for cessation of breastfeeding in case of breastfeeding complication	*p*
Mastitis	61.8	53.3	1.41 (1.33–1.50)	< 0.001	1.47 (1.38–1.57)	< 0.001
Medication for mastitis	61.6	52.8	1.43 (1.33–1.54)	< 0.001	1.50 (1.39–1.61)	< 0.001
Sore nipples	61.8	49.8	1.63 (1.53–1.75)	< 0.001	1.61 (1.50–1.73)	< 0.001
Other breastfeeding problems	63.0	33.6	3.36 (3.13–3.61)	< 0.001	3.22 (3.00–3.46)	< 0.001
Any breastfeeding problems	63.3	48.6	1.83 (1.75–1.91)	< 0.001	1.83 (1.74–1.92)	< 0.001

^a^Logistic regression adjusted for maternal age, maternal pre-pregnancy BMI, maternal education, family income, maternal smoking, fibre intake, and energy intake

Second, higher maternal education was associated with higher incidence of reported breastfeeding complications during the first month after delivery, as well as with longer duration of breastfeeding (see Supplemental Table 2).

## Discussion

In this population-based cohort study including 57,134 women, self-reported probiotic milk consumption during the first half of pregnancy was statistically associated with higher incidence of self-reported breastfeeding complications leading to healthcare consultations during the first month after delivery. Furthermore, self-reported probiotic milk consumption was associated with lower prevalence of breastfeeding cessation before the baby reached the age of 4 months.

The findings in this study do not support the hypothesis that general probiotic milk intake during pregnancy prevents future breastfeeding complications. However, results of different sensitivity analyses suggest that the association between probiotic milk intake and breastfeeding complications and duration might not be causal. First, no dose–response relationship between the amount of ingested probiotics and breastfeeding complications was found. Second, probiotics consumers breastfed longer despite increased incidence of reported breastfeeding complications. As in other studies [2, 25, 26], breastfeeding complications such as mastitis were associated with precocious weaning in this study population. Third, results seem to be confounded by socioeconomic factors such as education. Educated women reported a higher probiotic milk intake and are known to breastfeed longer [27]. At the same time, educated women have higher health literacy, defined as the capacity to obtain, process, and understand basic health information and services needed to make appropriate health decisions. They are, therefore, more observant and consult the health-care system more often [28, 29]. This might partly explain the higher prevalence of reported breastfeeding problems leading to healthcare consultations among probiotic milk consumers found in this study. However, results remained significant after adjustment for socioeconomic factors, as well as in stratified analysis for education, income, and BMI (results not shown).

This epidemiologic study underlines the need to perform RCTs with defined amounts of specified probiotic strains and clinical examination of the women.

While probiotic strains studied in this cohort were La-5, Bb12, and LGG*,* the RCTs previously performed investigated the effect of *Lactobacillus (L.) salivarius*, *L. gasseri,* and/or *L. fermentum.* A recently published study showed that a daily consumption of 250 ml product A as used in this study led to the presence of La-5, Bb12, and LGG in breast milk samples for only a small number of women [30]. If the positive effects of probiotics on breast health indeed should be accomplished by suggested local effects such as competitive exclusion [10], production of antimicrobials [11], normalization of breast tissue permeability [12], or increase of immunoglobulin A in breast milk epithelium [13], probiotic strains used in these commonly sold probiotic milk products might not be effective in regard to breastfeeding complications. However, another hypothesis links the risk for mastitis to the state of the host’s immune system [4]. Several RCTs based on product A were performed showing that intake of 250 ml product A/day during pregnancy and breastfeeding compared to a placebo fermented milk led to a higher prevalence of all three probiotic bacteria strains at 3 month postpartum in the mothers’ stool samples. Their children had a higher prevalence of LGG in their stool samples at 10 days and 3 months of age [31] and lower risk for atopic dermatitis at age 2 years [32]. Another RCT showed that ingestion of 250 ml/day of product A reduced the risk for antibiotic-associated diarrhoea [33]. Consumption of the probiotic milk consumed in this study might thus have an impact on the general immune state.

In 2008, Jiménez et al. randomized women (*n* = 20) with remaining mastitis symptoms after antibiotic treatment to intake of either *L. salivarius* and *L. gasseri* or placebo [7]. In 2010, Arroyo et al. randomized women (*n* = 352) with mastitis into three treatment groups: standard antibiotics, *L. fermentum,* or *L. salivarius *[6]. In both studies, the *Lactobacilli* groups had lower *Staphylococcus* counts after treatment and improved faster. Another RCT by the same group, published in 2016, evaluated a preventive effect of *L. salivarius* intake from pregnancy week 30 until delivery in women (*n* = 108) with a history of mastitis [8]. The probiotics group had a significantly lower incidence of mastitis and lower bacterial counts if mastitis did occur. In 2017, Hurtado et al. randomized women who were given antibiotic treatment at delivery (*n* = 625) to either intake of *L. fermentum* or placebo for 16 weeks. Women in the probiotic group had significantly lower incidence of mastitis [9]. Furthermore, this study differs by evaluating dietary intake during the first half of pregnancy even if it is assumed that the reported habitual intake of probiotic milk products in pregnancy is a proxy of the continued habitual intake during breastfeeding [16, 34]. In this epidemiologic setting, the outcome variables were based on the women’s self-reported data and their own initiative to use health-care services and not on medical records with International Classification of Disease (ICD) codes or examination performed by health-care professionals, which might have introduced bias as described above regarding level of education. Incidences of mastitis diagnosis and indications for antibiotic treatment differ considerably between different countries [1, 35], which further impedes comparison of study results from different countries. Another RCT on 8 week prophylactic *L. fermentum* intake is currently performed in Australia (*n* = 600) with a reported mastitis incidence of 15–21% comparable to the incidence in this population [36].

### Strengths and limitations

To the best of our knowledge, this is the first study to examine the possible effect of probiotic milk intake during pregnancy on breastfeeding complications and duration in an epidemiologic population-based setting. Strengths of this study are its size, with 57,134 women included, the comprehensive information on lifestyle and socioeconomics and the prospective design with registration of probiotic milk intake before possible breastfeeding complications might occur. The MoBa FFQ has been extensively validated [22, 37, 38]. However, several limitations need to be considered when interpreting the results. The outcome was based on the women’s self-reported data referring to if and why they contacted health-care services. As discussed above, this might have introduced bias, as better educated women are known to both breastfeed longer and having a lower threshold for contacting health-care services [28]. Even if a clinical follow-up of all women would have been desirable, it is unfeasible in a population-based study like MoBa. Since exposure is self-reported in a semi-quantitative FFQ, there is no information on the exact intake of probiotic bacterial count or measurement of actual bacterial count and type in the breast milk. However, the reported median daily intake in this study is comparable to the reported intake in most of the published RCTs [6-8]. The FFQ is answered at gestational week 22 and there is no comparable information on probiotics intake after delivery. However, it can be assumed that the reported habitual intake of probiotic milk products in pregnancy is a proxy of the continued habitual intake during breastfeeding. Although pregnancy is a time when most women think a lot about healthy eating, the major changes occur for intake of alcohol and coffee, while their core diet largely remains unchanged [34]. Maternal probiotic milk intake was also asked for in a less comprehensive way in MoBa Q1 and Q3 regarding the time period from before pregnancy to answering Q3 in pregnancy week 32, showing that most women continued to consume probiotic milk products as before pregnancy [16].

Administration of antibiotics, common as prophylaxis in obstetrics, or as treatment for manifest infection, might have interfered with the probiotic effect; this type of datum is not available in the MoBa data set. However, stratifying women by vaginal delivery or delivery by caesarean section, when prophylactic antibiotics treatment is usually given, did not change the results (data not shown). Unfortunately, MoBa provides no data on the exact time when the mother attracts breastfeeding complications or stops breastfeeding. Therefore, the possibility of reverse causality explaining the results—women breastfeeding longer having more time at risk for breastfeeding complications—cannot be completely excluded. However, as the analysis was restricted to women who initiated breastfeeding as well as to breastfeeding complications reported during the first month after delivery, we judge the risk for reverse causality as very low. Women were asked what, but not how, they fed their children; whether they were breastfeeding and/or bottle-feeding pumped breast milk might have affected the risk of developing breastfeeding complications. Despite adjustment for relevant confounders, residual confounding is probably still part of this association, as discussed above.

## Conclusions

Among 57,134 women from MoBa, self-reported probiotic milk intake during the first half of pregnancy was statistically associated with increased risk for self-reported breastfeeding complications, including mastitis. However, this association is probably not causal, as probiotic milk intake was also associated with longer breastfeeding duration. Further studies, specifically large RCTs with specified probiotic strains, defined exposure time, and clinical evaluation of breast complications in different populations, are needed to further investigate the association between probiotics intake and breastfeeding complications.

## Electronic supplementary material

Below is the link to the electronic supplementary material.

Supplementary file1 (DOCX 19 kb)
