# Supplementary material for: Maternal probiotic milk intake during pregnancy and breastfeeding complications in the Norwegian Mother and Child Cohort Study
Source: Eur J Nutr. 2019 Sep 10;59(5):2219–28. doi: 10.1007/s00394-019-02072-8 (PMC7351866; doi:10.1007/s00394-019-02072-8)
Supplement: Supplementary file 1 — Supplementary file1 (DOCX 19 kb) [file 394_2019_2072_MOESM1_ESM.docx]

Supplemental Table 1. Associations between categories of probiotics intake and breastfeeding complications and duration, n=57,134 women

|  | Non-consumers  (reference) | Low intake  OR (CI) | Moderate intake OR (CI) | High intake  OR (CI) | p for trend among probiotic consumers |
| --- | --- | --- | --- | --- | --- |
| Mastitis | 1 | 1.19 (1.09-1.30) | 1.10 (1.01-1.20) | 1.20 (1.08-1.32) | 0.98 ^a^ |
|  | 1 | 1.12 (1.03-1.23) | 1.03 (0.94-1.13) | 1.12 (1.02-1.24) | 0.72 ^b^ |
| Medication for mastitis | 1 | 1.21 (1.09-1.34) | 1.09 (0.98-1.21) | 1.12 (0.99-1.26) | 0.26 ^a^ |
|  | 1 | 1.14 (1.02-1.26) | 1.01 (0.91-1.13) | 1.06 (0.93-1.19) | 0.19 ^b^ |
| Sore nipples | 1 | 1.33 (1.21-1.47) | 1.33 (1.21-1.46) | 1.45 (1.31-1.62) | 0.21^a^ |
|  | 1 | 1.22 (1.10-1.35) | 1.18 (1.07-1.31) | 1.28 (1.15-1.42) | 0.46 ^b^ |
| Other breastfeeding problems | 1 | 1.27 (1.14-1.40) | 1.39 (1.27-1.53) | 1.56 (1.41-1.72) | 0.001 ^a^ |
|  | 1 | 1.14 (1.03-1.26) | 1.21 (1.10-1.33) | 1.33 (1.19-1.48) | 0.02 ^b^ |
| Any breastfeeding problems | 1 | 1.24 (1.16-1.33) | 1.25 (1.17-1.33) | 1.34 (1.24-1.44) | 0.12 ^a^ |
|  | 1 | 1.15 (1.08-1.24) | 1.13 (1.06-1.21) | 1.20 (1.11-1.30) | 0.50 ^b^ |
| Cessation of predominant breastfeeding before 4 months | 1 | 0.94 (0.89-0.99) | 0.86 (0.82-0.91) | 0.87 (0.82-0.92) | 0.02 ^a^ |
|  | 1 | 1.00 (0.95-1.06) | 0.92 (0.88-0.97) | 0.91 (0.86-0.97) | 0.005 ^b^ |
| Cessation of any breastfeeding before 4 months | 1 | 0.67 (0.62-0.73) | 0.59 (0.54-0.64) | 0.62 (0.57-0.69) | 0.18 ^a^ |
|  | 1 | 0.82 (0.75-0.89) | 0.76 (0.69-0.83) | 0.80 (0.73-0.89) | 0.61 ^b^ |

Logistic regression: ^a^ first row for each variable unadjusted, ^b^ second row adjusted for maternal age, maternal pre-pregnancy BMI, maternal education, family income, maternal smoking, fibre intake, energy intake, non-probiotic yoghurt consumption and non-probiotic milk consumption

Supplemental Table 2. Breastfeeding complications and duration according to maternal education, numbers (% of respective category of maternal education), n=57,134 women.

|  | ≤12 years | 13-16 years | >16 years | missing |  |
| --- | --- | --- | --- | --- | --- |
|  | n=16,747 | n=24,396 | n=14,872 | 1,119 | p[[28](#_ENREF_28)]^1^ |
| Mastitis | 1,192 (7.1) | 2.079 (8.5) | 1,334 (9.0) | 70 (6.3) | <0.001 |
| Medication for mastitis | 745 (4.4) | 1,416 (5.8) | 921 (6.2) | 45 (4.0) | <0.001 |
| Sore nipples | 848 (5.1) | 1,540 (6.3) | 1,141 (7.7) | 66 (5.9) | <0.001 |
| Other breastfeeding problems | 848 (5.1) | 1,578 (6.5) | 1,170 (7.9) | 69 (6.2) | <0.001 |
| Any breastfeeding problems | 2,202 (13.1) | 3,852 (15.8) | 2,582 (17.4) | 153 (13.7) | <0.001 |
| Predominant breastfeeding for four months | 8,776 (52.4) | 15,480 (63.5) | 9,979 (67.1) | 664 (59.3) | <0.001 |
| Any breastfeeding for four months | 13,227 (79.0) | 22,252 (91.2) | 14,119 (94.9) | 969 (86.6) | <0.001 |

^1^P-value according to Pearson’s chi-square test.
